# Seed dispersal of *Diospyros virginiana* in the past and the present: Evidence for a generalist evolutionary strategy

**DOI:** 10.1002/ece3.3008

**Published:** 2017-05-04

**Authors:** Mimi Rebein, Charli N. Davis, Helena Abad, Taylor Stone, Jillian del Sol, Natalie Skinner, Matthew D. Moran

**Affiliations:** ^1^ Department of BiologyHendrix CollegeConwayARUSA; ^2^ Present address: Department of BiologyGilbert HallStanford UniversityStanfordCA94305‐5020USA; ^3^ Present address: Division of Biological Sciences (DBS)University of Montana32 Campus Dr. HS 104MissoulaMT59812USA

**Keywords:** anachronism, Anthropocene, *Diospyros virginiana*, mesopredators, paleoecology, persimmon, seed dispersal

## Abstract

Several North American trees are hypothesized to have lost their co‐evolved seed disperser during the late‐Pleistocene extinction and are therefore considered anachronistic. We tested this hypothesis for the American persimmon (*Diospyros virginiana*) by studying the effects of gut passage of proposed seed dispersers on seedling survival and growth, natural fruiting characteristics, and modern animal consumption patterns. We tested gut passage effects on persimmon seeds using three native living species, the raccoon (*Procyon lotor*), Virginia opossum (*Didelphis virginiana*), and coyote (*Canis latrans*), and two Pleistocene analogs; the Asian elephant (*Elephas maximus*) and alpaca (*Vicugna pacos*). Persimmon seeds excreted by raccoons, coyotes, and elephants survived gut transit. Gut passage did not affect sprouting success, but did tend to decrease time to sprout and increase seedling quality. Under field conditions, persimmon fruits were palatable on the parent tree and on the ground for an equal duration, but most fruits were consumed on the ground. Seven vertebrate species fed upon persimmon fruits, with the white‐tailed deer (*Odocoileus virginianus*)—a species not capable of dispersing persimmon seeds—comprising over 90% of detections. Conversely, potential living seed dispersers were rarely detected. Our results suggest the American persimmon evolved to attract a variety of seed dispersers and thus is not anachronistic. However, human‐induced changes in mammal communities could be affecting successful seed dispersal. We argue that changes in the relative abundance of mammals during the Anthropocene may be modifying seed dispersal patterns, leading to potential changes in forest community composition.

## 1 Introduction

Several North American plants are hypothesized to be anachronistic (Barlow, 2001), in that they lack living seed dispersers (Janzen, 1982; Janzen & Martin, 1982). It has been hypothesized that the end‐Pleistocene extinction event in North America—particularly the disappearance of numerous large mammals—resulted in the loss of co‐evolved plant–animal relationships. For instance, several trees have fruits that offer a high potential energy reward, yet appear to have no living dispersers (Barlow, 2001). This loss of potential seed dispersers may have affected the distance seeds travel from parent plants and may explain why the realized range (without human assistance) was often smaller than the potential range of these species (Berry, 1916; Burton, 1990; Murphy, 2001; Skallerup, 1953). For example, the Osage orange (*Maclura pomifera* Raf.) was historically found (at the time of European settlement) in small areas of Texas, Oklahoma, and Arkansas, but today it has spread by human dispersal throughout most of temperate North America (Barlow, 2000). Some species (squash, *Curcubita* spp.) may even have been on a pathway to extinction in the post‐Pleistocene world because of their reduction in seed dispersal, but survived through domestication by aboriginal humans (Kistler et al., 2015).

The scope of possible large mammal seed dispersers during the Pleistocene is extensive. It has been suggested that nonruminant herbivores, such as mastodons, mammoths, gomphotheres, horses, and giant ground sloths, because of their high mobility and relatively mild guts, would have made particularly effective seed dispersers (Boone et al., 2015; Janzen & Martin, 1982). Today, there are no surviving large, nonruminant mammals in temperate North America, with the exception of the collard peccary (*Pecari tajacu* L.) in the southwest United States. Ruminant mammals also suffered high Pleistocene extinction rates, but still exist in moderate diversity throughout temperate parts of the continent. However, ruminants are largely considered poor seed dispersers for many plant species because of their complex digestive process (Barlow, 2000; Cosyns, Delporte, & Hoffman, 2005; Prasad, Krishnaswamy, Chellam, & Goyal, 2006; but see Janzen, 1982).

Experimental studies indicate that the seeds of some proposed anachronistic fruits sprout and produce higher quality seedlings after passing through the guts of Pleistocene ecological analogs (e.g., Asian Elephant, *Elephas maximus* L., Boone et al., 2015), while others have shown that native extant mammals could also be effective seed dispersers for these plants (Cypher & Cypher, 1999; Roehm & Moran, 2013). Several studies indicate that animals of the Order Carnivora, which often consume large amounts of fruit, may be the important dispersers for fruits that are considered anachronistic (Bustamante, Simonetti, & Mella, 1992; González‐Varo, López‐Bao, & Guitián, 2013; Koike, Morimoto, Goto, Kozakai, & Yamazaki, 2008; Willson, 1993). For many proposed anachronistic plant species, however, there is little information on current fruit consumption by extant animals and there are few experimental studies on gut passage effects on survival, sprouting, and seedling growth rates.

**Figure 1:**
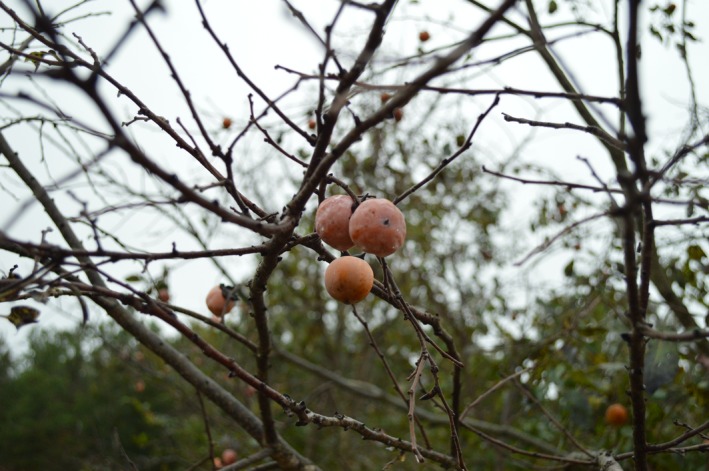
The American persimmon (*Diospyros virginiana*) fruit

The American persimmon (*Diospyros virginiana* L.) has fruits described as “moderately anachronistic” (Barlow, 2001). The fruit is large (2–6 cm diameter, Figure 1), fleshy, sweet, and offers high caloric value (USDA, 2016a). There is little information on which animal species contribute to persimmon fruit consumption and seed dispersal rates in the wild, and there are no records of persimmon seed dispersal by obligate herbivorous mammals. The fruit is also too large for almost any bird to swallow, although several species are known to consume the pulp of the fruit (Skallerup, 1953). It is readily eaten by multiple species of Carnivora (and one omnivorous marsupial), although there is conflicting evidence about seed dispersing effectiveness of these species (Boone et al., 2015; Chavez‐Ramirez & Slack, 1993; Cypher & Cypher, 1999; Everitt, 1984; Martin, Zim, & Nelson, 1951; Roehm & Moran, 2013; Worth, 1975). So while the fruit of *D. virginiana* has some characteristics of an anachronism, observational and experimental studies testing this hypothesis are limited.

To test the hypothesis that the American persimmon is anachronistic, we performed replicated controlled field experiments where we measured the sprouting success and quality of plants produced from persimmon seeds that had passed through the digestive system of a variety of hypothesized past and current dispersers. These species included native extant small/medium sized mammals and large mammals that represented analogs of extinct Pleistocene species. We also measured the animal visitation rates at individual persimmon trees, fruit ripening and palatability patterns, and fruit consumption rates under natural conditions. The experimental field studies were designed to compare seed dispersal success of modern species compared to extinct potential dispersers, while the observational studies were designed to determine how the modern animal fauna affects potential seed dispersal success. If *D. virginiana* has anachronistic fruit, we predict higher sprouting success, higher seedling survival, and/or higher seedling quality for those seeds that passed through the guts of our Pleistocene analogs while field observations would indicate poor dispersal rates.

## 2 Materials and Methods

### 2.1 Study site

We conducted our observations and field experiments in Conway, Perry, and Faulkner counties of central Arkansas. In this region, the American persimmon (*D. virginiana*) is fairly common in disturbed areas and along forest‐pasture boundaries. The native vegetation in the region is mixed oak/hickory/pine forest. Most native mammals are present, with the exception of wolves (*Canis lupus* L.), elk (*Cervis canadensis* Erxleben), and bison (*Bison bison* L.). The region is mostly rural—with native forest and pastures covering ~80% of the landscape in roughly equal amounts—and smaller amounts of row crops and developed areas (Moran, Cox, Wells, Benichou, & McClung, 2015).

### 2.2 Seed survival and growth field experiments

We tested the germination rates, seedling survival, and seedling growth rates for seeds that passed through the digestive system of three native mammals and two Pleistocene analogs. In modern times, the mesopredator community has been considered the most likely disperser of persimmons (Cypher & Cypher, 1999; Martin et al., 1951; Skallerup, 1953). During the Pleistocene, elephants (e.g., mastodons, *Mammut* spp.), horses (*Equus* spp.), and camels (several genera) were some of the most abundant megafauna in the southeast United States, and therefore likely fed upon persimmons. We had access to modern analogs for each of these extinct groups of animals using Asian Elephants (*Elephas maximus*) and alpacas (*Vicugna pacos* L.) for Mastodons and American camels, respectively. Previous studies demonstrated that horses readily consume persimmon fruits, but the seeds do not survive gut passage (Boone et al., 2015), so we excluded this Pleistocene analog from our study. For extant native species, we tested the coyote (*Canis latrans* Say), raccoon (*Procyon lotor* L.), and Virginia opossum (*Didelphis virginiana* Kerr), three of the most common mesopredators found in our study region. We collected seeds from dung in captive elephants, opossums, and alpacas, while seeds were collected from dung in the natural environment for raccoons and coyotes since they were unavailable in captivity. On 24 October 2014, we fed two female Asian elephants 50 persimmons each. On 22 October 2015, we provided a group of six alpacas a total of 75 ripe persimmons. On 19 October 2015, we fed four opossums four fruits each. For each of the captive animals, we collected dung for 48 hr post‐feeding, sorted it and recovered persimmon seeds if present. This time period is beyond the normal gut passage time for all species (Hackenberger, 1987; Van Weyenberg, Sales, & Janssens, 2005). We collected seeds from dung in the wild on 15–25 October 2015 and 16–27 October 2014 for raccoons and coyotes, respectively. Raccoon dung containing persimmon seeds was scarce in 2014, but more common in 2015, and we only had access to opossums and alpacas during 2015, which necessitated conducting these experiments in separate years.

On 1 November 2014, we initiated a field experiment testing the sprouting success and growth rates of persimmon seeds that had passed through the guts of the elephants and coyotes compared with manually dissected seeds and whole fruit. The dissected seed and whole fruit treatments were controls designed to test seed removal from fruit (similar to what occurs during animal digestion) compared to potential fruit inhibitory effects on sprouting and growth (Samuels & Levey, 2005). Fifty replicates of each treatment were intentionally interspersed in an 8 × 25 array, each planted 0.5 m apart, at a depth of 2.5 cm and then uniquely labeled with a flag. We estimated the number of seeds in the whole fruits treatment by averaging the number of seeds present in 30 laboratory dissected whole fruits. We enclosed the array in a 0.9‐m‐high wire mesh fence to prevent disturbance by nine‐banded armadillos (*Dasypus novemcinctus* L.), which are common in the area and tend to forage in disturbed soils M.D. Moran personal observation). The same design was initiated on 3 November 2015 using seeds collected from raccoon scat, dissected seeds, and the whole fruit treatments. Alpacas and opossums did not excrete viable seeds, so were not included the field experiments (see Results).

After the winter cold stratification periods, we monitored the arrays daily for sprouts. In order to prevent competition from the multiple seedlings that emerged from the whole fruit treatments, we removed all sprouts after the first seedling emerged, so that we had at most one plant per replicate for all treatments. Four times during the growing season, early June, early July, early August, and late August (exact dates varied slightly between the 2014, 2015, and 2015, 2016 experiments), we measured three variables as indicators of plant quality and growth: plant height, number of leaves, and stem diameter at ground level. At the end of the experiment, we clipped all surviving seedlings at ground level, dried them at 50°C for 24 hr, and weighed them to determine dry mass.

### 2.3 Faunal observation and fruit fate study

To determine current potential seed dispersers of *D. virginiana*, we observed the faunal community that feeds on the fruit of this species in the natural environment. In the autumn of 2014, we selected four mature persimmon trees on Petit Jean Mountain, Conway County, AR. We installed a single wildlife camera (Wildgame Innovations™, New Roads, LA, USA, Model #i10b38d2) 10 m from the tree in question, facing the tree with field of view covering the trunk and ground area where the fallen persimmons were located. All trees selected were in open areas that did not touch the crowns of other trees so that we could detect animals feeding on the ground or climbing the trees, although the field of view was too small to capture the entire trees and birds that may have flown into the trees to feed. Cameras were active from 14 October 2014 to 8 November 2014, corresponding to the time when mature fruit was present and resulted in 2,496 total camera hours. Each camera was equipped with a motion detector and infrared camera to photograph animals automatically. Photographs from the camera were downloaded daily. While this method does not directly indicate which species consumed individual fruits, we assume that detection frequency of foraging species is an effective indicator of consumption rates. In many cases, however, we photographed animals consuming persimmon fruits. To determine species‐specific animal visitation rates, we calculated detection frequency as the number of frames in which a particular species was present during the course of the study. We initially planned to monitor another four trees in the autumn of 2015, but that year very few persimmon trees successfully produced fruit, perhaps because of record rainfall anomalies during the spring flowering period (National Weather Service, 2015).

To determine duration of time when persimmon fruits were edible, we tracked 45 fruits that were attached to the parent trees (six different trees) and 45 fruits that were on the ground and protected from consumption. Before ripening, each fruit was uniquely labeled with a Sharpie™ pen and tracked from ripening until abscission, or from time on the ground until rotting. A fruit was considered ripe when the color turned dark orange, and the fruit was soft to the touch. Previous studies indicate that this point is when persimmon fruits lose their astringency and become edible (Taira, 1996). Rotten fruits were classified as those that turned a dark purple/black color and shriveled. The subjective measure is somewhat arbitrary, but could be confirmed by consumption patterns over time (see next observational study).

For the fruits attached to the parent tree, we also attempted to locate marked fruit that had fallen from the tree to determine whether they had been consumed on the tree or simply abscised. As fallen fruits may have been consumed by a ground forager before we could recover them (if consumed between daily checks), we could only calculate minimum on‐tree survival.

To determine consumption rates once abscised, we marked 10 ripe persimmons on the ground at each of the four photographed trees. If a persimmon fruit was consumed by an animal, we labeled a new fruit, so that at any one time, we were tracking 10 individual fruits per tree (*N* = 292 total). We followed fruits from the beginning of abscission until all fruits on the trees had either fallen or rotted (14 October 2014 to 8 November 2014), using the definition of rotten from the previously described observations.

We recognize that because we monitored multiple fruits on individual trees, there is inherent and unavoidable pseudoreplication in the design of the faunal observations and fruit fate studies, limiting the representative nature of the sample and the interpretation of the data. However, there were not enough individual trees available (nor was it logistically possible) at our field sites to design a fully replicated study. Therefore, abscission rates, consumption (disappearance) rates, and palatability times were plotted and analyzed using the mean value from each tree as a replicate.

### 2.4 Analysis

For the field experiments testing seed disperser species effects on seedling quality, we compared sprout success and survival after sprouting by a chi‐square analysis. As the number of leaves, stem diameter, and stem height were all measures of plant quality, we analyzed these data by repeated measures MANOVA, followed by individual repeated‐measures ANOVA if the between subjects MANOVA was significant. The number of days to sprout and final plant mass was analyzed individually by a one‐way ANOVA. All post hoc analyses were performed using a Tukey test.

In the observational studies, the proportional number of fruits consumed (i.e., disappeared) while attached to persimmon trees versus proportion consumed on the ground and the comparison of palatable days were analyzed by a *t* test of independent samples (the mean of each tree was a replicate to account for pseudoreplication). As there was no statistical comparison to perform on the animal visitation rates, these data were presented as the mean number of animals per species per tree (±1 *SD*). Fruit consumption and abscission patterns were presented as age‐specific rates (i.e., each day was one age class) over time.

## 3 Results

### 3.1 Fruit feeding and plant quality field experiments

Asian elephants, alpacas, and Virginia opossums (the three captive species) readily consumed persimmons in our experimental feeding trials. Elephants and alpacas completely consumed the persimmon fruits, including the seeds. Virginia opossums consumed fruits, but were observed expelling the seeds without swallowing them. Previous research found that about 50% of the seeds consumed by elephants survive gut passage intact (Boone et al., 2015). Although we did not monitor the number of seeds in these experiments, we found numerous intact seeds in the elephant dung. We found no intact seeds in alpaca dung, although we did find remains of persimmon fruit (bright orange discolorations) and many seed fragments. We collected numerous seeds that had been expelled from the mouths of opossums as they consumed the fruit, but we only found one seed in the dung (of an estimated 52 present in the fruit at feeding time). The raccoon and coyote dung we collected in the field almost invariably had large numbers of intact persimmon seeds present.

**Figure 2:**
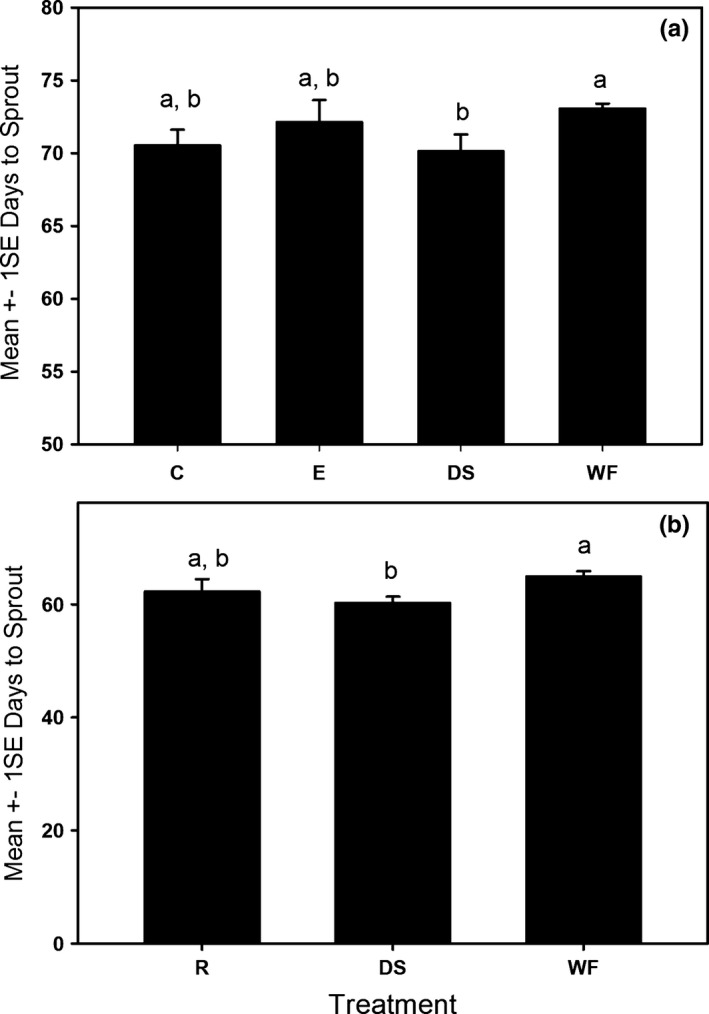
Mean days to sprout comparison between treatments in field experiments in (a) 2015 and (b) 2016. C, coyote; E, elephant; DS, manually dissected; WF, whole fruit; R, raccoon. Letters indicate significantly different subgroups (Tukey).

The survivorship and growth field experiments showed differences between years independent of treatment that were probably caused by weather variation. For instance, seeds sprouted faster in the 2015–2016 compared to 2014–2015 (Figure 2). There was no significant difference in the proportional number of seeds sprouting for any treatment in either experiment (Table 1). We also found no significant postsprouting survivorship differences for either experiment (Table 1). Number of days to sprout was significantly different between treatments in 2014–2015 (one‐way ANOVA, *F*_4,353_ = 3.95, *p* = .004, Figure 2a) and 2015–2016 (one‐way ANOVA, *F*_2,185_ = 3.72, *p* = .03, Figure 2b) experiments. According to the post hoc analysis, in both experiments, the dissected seed (DS) treatment was significantly faster to sprout compared to the whole fruit (WF) treatment (Figure 2a,b). The three animal treatments sprouted at rates that were intermediate and not significantly different from either the DS or WF treatments. Although we did find significance in the days to sprout, it should be noted that the effect size was small (<5% difference between the smallest and largest treatment means).

**Table 1:** Results of seeds sprouting and proportion of seedlings surviving postsprouting in 2015 and 2016 field experiments testing gut passage of native and Pleistocene analog species

	R	E	C	DS	WF	χ^2^	*p*
2015 Experiment							
Initial seed number	—	50	50	50	324 (estimated)		
Proportion sprouting	—	0.76	0.78	0.88	0.79	2.67	ns
Initial sprout number	—	38	39	44	45		
Proportion surviving	—	0.76	0.74	0.75	0.86	2.58	ns
2016 Experiment							
Initial seed number	50	—	—	50	208 (estimated)		
Proportion sprouting	0.68	—	—	0.68	0.56	4.28	ns
Initial sprout number	34	—	—	34	38		
Proportion surviving	0.88	—	—	0.71	0.79	3.23	ns

R, raccoon; E, elephant; C, coyote; DS, dissected seeds (manually from fruit); WF, whole fruit.

**Figure 3:**
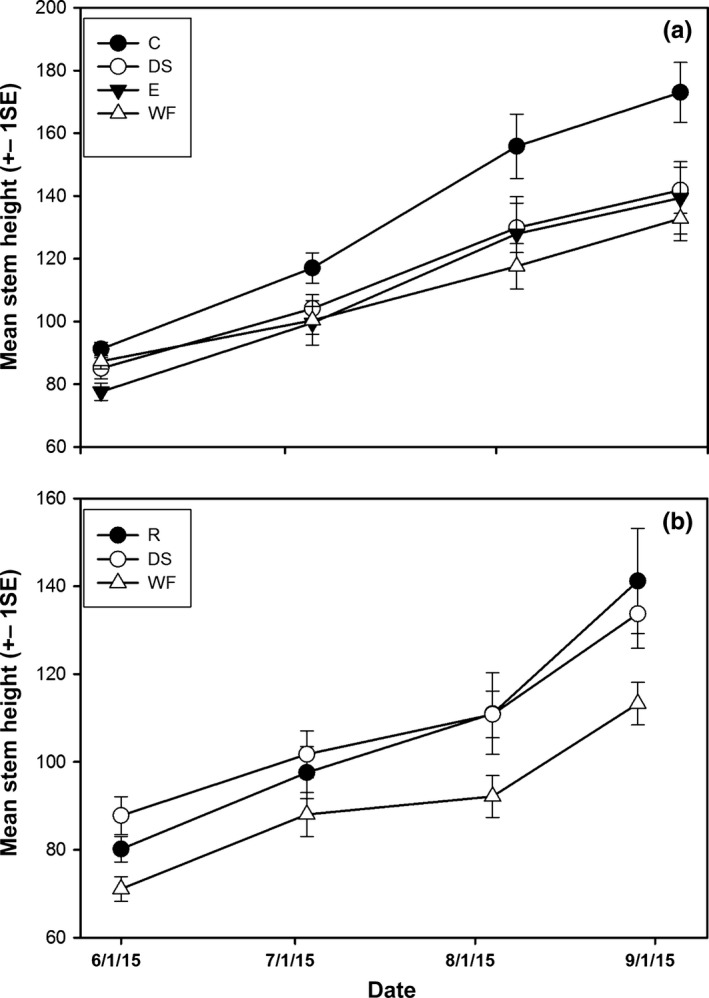
Mean stem height comparison between treatments in field experiments in (a) 2015 and (b) 2016. C, coyote; E, elephant; DS, manually dissected seeds; WF, whole fruit; R, raccoon

**Figure 4:**
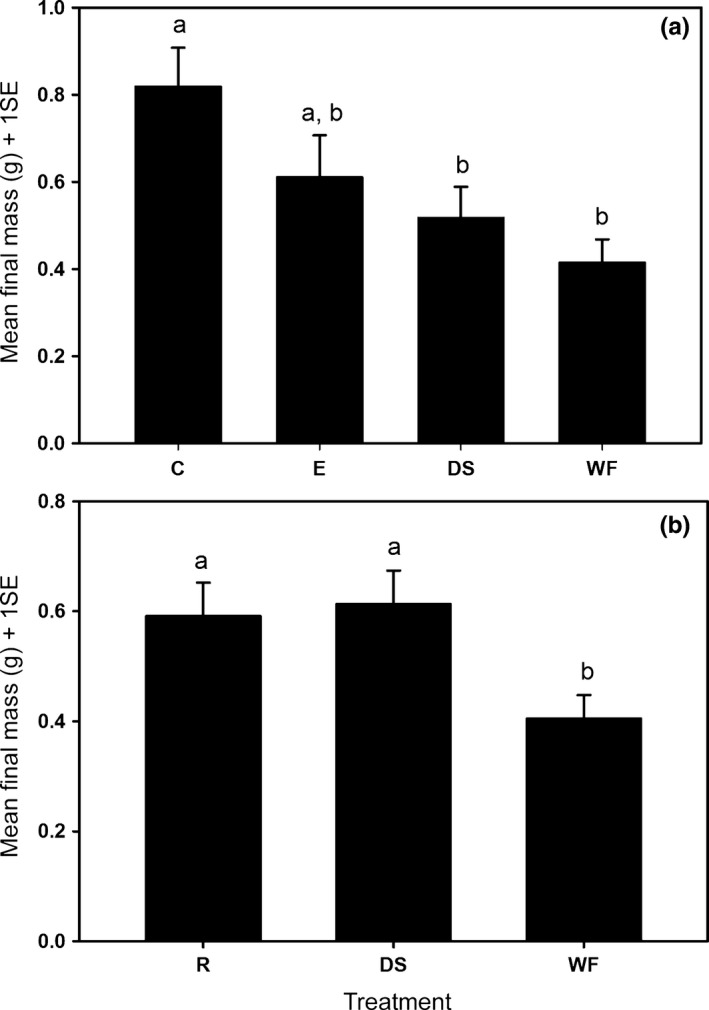
Mean final mass comparison between treatments in field experiments in (a) 2015 and (b) 2016. C, coyote; E, elephant, DS, manually dissected seeds; WF, whole fruit; R, raccoon. Letters indicate significantly different subgroups (Tukey).

In the 2014–2015 field experiment, there was a significant effect of treatment on plant quality values that we measured multiple times (leaf number, stem height, and stem diameter, MANOVA, Wilks' λ = 0.869, *F*_9,292_ = 1.94, *p* = .048). For the individual traits, we found no significant difference between treatment groups for mean number of leaves per plant (repeated measures ANOVA, *F*_3,122_ = 1.47, *p* = .23) or stem diameter (log_10_‐transformed data, *F*_3,122_ = 2.30, *p* = .08), but the stem heights were significantly different (*F*_3,122_ = 3.42, *p* = .02, Figure 3a) with the coyote treatment taller than all other groups. In the 2015–2016 field experiment, there was a significant effect of treatment on plant quality values we measured multiple times (MANOVA, Wilks' λ = 0.743, *F*_6,148_ = 3.95, *p* = .001). For the individual traits, we again found no significant difference between treatment groups for mean number of leaves per plant (repeated‐measures ANOVA, *F*_2,76_ = 1.73, *p* = .18) or stem diameter (repeated measures ANOVA, *F*_2,76_ = 2.23, *p* = .14). However, mean stem height was significantly taller for the raccoon and dissected seed treatments compared to the whole fruit treatment (ANOVA, *F*_2,76_ = 4.39, *p* = .02, Figure 3b). We found that the dry mass of persimmon seedlings was significantly different in the 2014–2015 experiment, with the coyote seedlings the heaviest, the whole fruit seedlings the smallest, while the elephant and dissected treatments of intermediate mass (one‐way ANOVA, *F*_3,126_ = 5.25, *p* = .002, Figure 4a). In the 2015–2016 experiment, final dry mass of persimmons seedlings from seeds that had passed through raccoons and those that were manually dissected were significantly larger than those sprouting from whole fruits (one‐way ANOVA, *F*_2,78_ = 4.53, *p* = .01, Figure 4b).

### 3.2 Fauna, fruit ripening, and fruit consumption observations

During the 2,496 camera hours, we obtained 494 animal detections that included five genera (six species) of mammals and one bird species (See Fig. S1 for examples). Of these seven species visiting the persimmon trees, white‐tailed deer (*Odocoileus virginianus* Zimmerman) made up 93% of all detections, with the other six species comprising a small proportion of animals feeding on the fruits (Table 2). Surprisingly, we had no coyotes (*C. latrans*) visit the fruiting sites, although they are commonly seen in the area.

**Table 2:** Detection frequency and proportional representation of vertebrates feeding on *D. virginiana* fruit. Values are mean (±1 *SD*) per tree

Species	*Odocoileus virginianaus*	*Corvus brachyrhynchos*	*Sciurus* spp.	*Vulpes vulpes*	*Procyon loter*	*Didelphis virginiana*
Mean (±1 *SD*)	114.5 (34.8)	3.5 (5.7)	2.0 (3.4)	1.8 (3.5)	1.3 (2.5)	0.5 (1.0)
Proportion	0.93	0.03	0.01	0.01	0.01	<0.01

**Figure 5:**
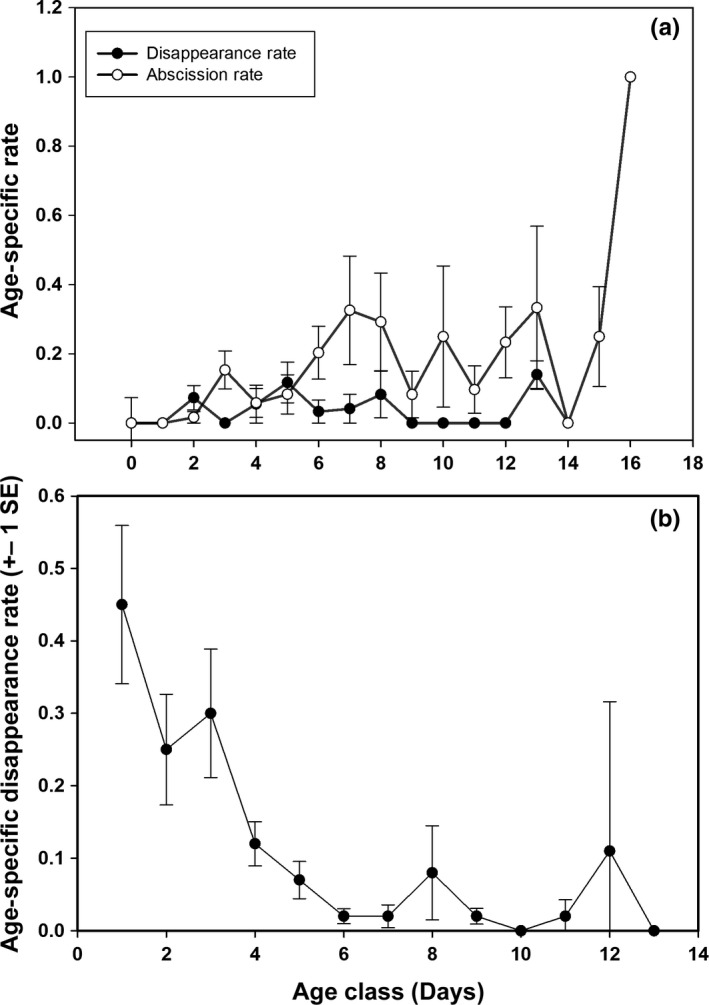
Age‐specific (a) disappearance and abscission rates for persimmon fruits attached to parent tree and (b) disappearance rates on the ground. Values are rate (±1 *SE*) on a per tree basis

Persimmons were ripe and presumably palatable for about 8 days, both on the tree (7.85 ± 0.52 days) and on the ground (7.74 ± 0.82 days), with no significant difference in palatable time period (*t* = 0.13, *p* = .93). More of our labeled persimmons on the ground disappeared (0.73 ± 0.10 proportion per tree) and were presumably consumed by the animals photographed, while a much smaller proportion attached to the tree disappeared (0.24 ± 0.03, *t* = 14.7, *p* = 0.001). As fruits could fall from the tree and be consumed on the ground between our daily monitoring, disappearance rates on the tree may be an overestimate, that is, they may have fallen but were consumed on the ground before the daily monitoring occurred. However, we consider the ground disappearance rate a more reliable estimate of the number consumed by potential seed dispersers. Therefore, the large difference in consumption rates on the ground compared to on the tree is probably higher than we observed. Once on the ground, the disappearance rate was high during the first 3 days (Figure 5b), after which few disappeared before rotting. No persimmons were eaten from the ground after day 13. For persimmon fruits attached to the parent tree, there was no pattern to the disappearance rate (i.e., it was low each day), while the abscission rate increased after day five postripe. No fruit remained on the tree more than 16 days postripe (Figure 5a).

## 4 Discussion

The survivorship and plant quality field experiments demonstrated that a variety of mammals are potential seed dispersers for the American persimmon. Seeds that had passed through the digestive system of raccoons and coyotes, as well as seeds that were manually dissected from the fruits, generally produced higher quality plants compared to those that sprouted from whole fruits. The seeds that had passed through the elephant digestive system tended to be of higher quality, although this trend was nonsignificant. In all experiments, survivorship was unaffected by treatment, so gut passage only affected plant quality. Interestingly, these results differ from previous greenhouse experiments where persimmon seeds sprouted at much higher rates if consumed by elephants compared to whole fruit, but plant quality was similar (Boone et al., 2015), while seeds that had been consumed by coyotes had lower seedling quality compared to whole fruits (Roehm & Moran, 2013). These results show the possible effects of field versus greenhouse experiments. In both greenhouse studies, experiments were undertaken during the winter when greenhouse humidity levels were reduced due to artificial heating. We suggest this condition may have affected sprouting success in whole fruits by reducing the rotting rate. We consider the field experiments in this study to be more representative of natural conditions where seeds sprouted under high humidity conditions that are common in the springtime of the mid‐south.

Seeds that were manually dissected, but did not pass through the digestive system of an animal, tended to have the same quality as seeds that had undergone gut passage. Interestingly, this result suggests that simply being removed from the fruit may be the most important factor for seedling growth. Many fruits have compounds that inhibit seed sprouting and growth (Robertson, Trass, Ladley, & Kelley, 2006), a probable adaptation to delay sprouting until dispersal. These results thus indicate that the fruit of the American persimmon evolved to attract a variety of dispersers and was not co‐evolved with a single animal species, as hypothesized by some researchers (Barlow, 2000). Our results support the notion that animal dispersed fruits usually reflect a generalist strategy (Howe & Smallwood, 1982), which appears to commonly evolve in temperate ecosystems (Willson, 1993).

Our results also support the notion that some extinct Pleistocene megafauna, notably elephants, could have dispersed this plant effectively (Boone et al., 2015). Indeed, intact persimmon seeds have been found in mastodon dung at one fossil site (Newsom & Mihlbachler, 2006). Large Pleistocene mammals could have been highly effective dispersers (although not obligatory) because they would have presumably traveled long distances and been capable of consuming large quantities of fruit. Unfortunately, the limited fossil record of gut contents for mastodons and other Pleistocene mammals from temperate habitats (there are more numerous remains from animals in colder climates, e.g., woolly mammoths, van Geel et al., 2008) does not allow us to determine the ubiquity of this feeding behavior. While seed dispersal in the Pleistocene has often been speculative (Barlow, 2000), we argue that performing paleoecological experiments such as these can elucidate past ecological and evolutionary relationships.

Based on the detection frequency of potential seed dispersers, our camera data show that *O. virginianus* (white‐tailed deer) was probably the major consumer of persimmon fruit, while several other species consumed much smaller quantities. However, white‐tailed deer are not effective seed dispersers of plants such as persimmons. As white‐tailed deer are ruminants that chew their cud and ferment their food for long periods of time, it is highly unlikely that the large seeds of persimmons survive gut passage. Furthermore, there are no records of deer scat with intact persimmon seeds, nor any seeds of that size, and our observations of hundreds of deer scats over the years at these field sites have never shown a single intact persimmon seed (personal observation). Additionally, Myers, Vellend, Gardescu, and Marks (2004) reported many small viable seeds in deer dung, but none greater than 7 mm in diameter (less than 50% the size of typical persimmon seed). Therefore, we are confident in our assessment that although deer consume persimmon fruit at a high rate, the seeds are not dispersed by this species.

The other consumers of persimmon fruit were mesopredators (raccoons, foxes, and opossums), one bird species (American crow *C. brachyrhynchos* Brehm), and two species of squirrels (*Sciurus* spp.). Raccoons and coyotes have been recorded feeding on persimmon fruits (Martin et al., 1951) and successfully dispersing viable seeds (Cypher & Cypher, 1999; Everitt, 1984). However, raccoons were relatively rare and coyotes were absent from our camera data (although the number of trees samples is admittedly small). The absence of coyotes at persimmon trees was particularly surprising, given that practically all coyote dung observed during our study contained persimmon seeds (personal observation). Additionally, coyotes are common at the persimmon field sites and have frequently been captured on camera data from other experiments. So it appears that although individual coyotes frequently feed on persimmons, their density is much lower compared to other animals feeding on the fruits. Although crows and squirrels were recorded feeding on persimmons, it is unclear whether they are effective seed dispersers. Our personal observations indicated that crows consume the fruit pulp but do not swallow the seeds, while squirrels have not been recorded to cache persimmon seeds (Skallerup, 1953) in the manner they do many other seeds (e.g., oak acorns, Fox, 1982). However, if these animals carry fruit to distant sites before consuming it, especially likely for very mobile animals like crows, then they may be valuable dispersers of persimmon seeds. Opossums, although captured by cameras in small numbers at the persimmon trees, do not consume persimmon seeds, according to our opossum feeding trial using captive animals (see Results). However, this result is in direct conflict with observations made by Worth (1975), who recorded opossum dung with numerous persimmon seeds under field conditions. We also recorded very small numbers of red fox (*Vulpes vulpes* L.), which we do view as a potentially successful seed disperser, as it is also a small Carnivora, similar in size to the raccoon, and known to be an effective seed disperser of a variety of plant species (Bustamante et al., 1992; Martin et al., 1951). Wild Turkey (*Meleagris gallopavo*) would also seem to be a potential consumer of persimmon, but although they are common in our study area, they did not appear at any of our camera sites. Additionally, feeding records of wild turkeys do not include persimmons (Dalke, Clark, & Korschgen, 1942; Eaton, 1992; Meanley, 1956), perhaps because they are focused on hard mast crops (e.g., acorns) during the autumn period when persimmons are ripe. Therefore, in terms of animals capable of successfully dispersing seeds, we detected several species, but they comprised a very small percentage (about 2%) of our camera detections.

The results of the fruit ripening observations show that edible fruit is present on the tree and on the ground for almost the exact same amount of time. However, there appears to be very little consumption by animals climbing (or flying into) the trees to acquire fruit. Our cameras never captured any animals climbing the trees, perhaps because the fruit is usually found on the ends of the branches that cannot support the weight of a mammal. We have occasionally observed American crows (*C. brachyrhynchos*) landing in persimmon trees. As very little fruit disappeared while attached to the tree, but it disappeared rapidly while on the ground, we argue that presently, persimmon fruit is commonly consumed only after abscission. In the past though, there is one conspicuous animal group that could have fed upon the fruit while still attached and successfully dispersed the seeds—North American elephant species (e.g., *M. americanum* Kerr). Most persimmon trees are relatively short (less than 7 m, USDA, 2016b), so fruit could have easily been reached by a mastodon‐sized animal, although they could have certainly fed on fallen fruit as well.

Many factors are currently affecting the community structure of North American forests including disease, pollution, and introduced species (Lovett, Canham, Arthur, Weathers, & Fitzhugh, 2006; Percy & Ferretti, 2004). While not discounting these issues, our observations and field experiments show that the human‐induced change in extant faunal communities could also affect seed dispersal of native flora. White‐tailed deer overabundance has been implicated in substantial changes in forest community structure, nutrient cycling, and successional patterns (Côté, Rooney, Tremblay, Dussault, & Waller, 2004). While their browsing of vegetation is usually invoked to explain changes in forest dynamics, their effects on seed dispersal have been less well studied (Gill & Beardall, 2001). We suggest that their large increase in population size could be reducing seed dispersal for some species, especially those plants with large seeds adapted for dispersal by nonruminant mammals. Similarly, coyotes have been widely observed feeding on persimmon fruit and dispersing the seeds. However, this species was not originally found over most of the range of *D. virginiana*. In the southeast USA, the native canid was the red wolf (*C. rufus* Audubon and Bachman). However, we suspect that the dispersal ability of these two species was similar as they are very closely related, and *C. rufus* has been observed feeding on persimmons (and seeds have been observed in its dung) in the small area of North Carolina where it has been reintroduced (David Rabon, personal communication).

Therefore, it is not just the faunal species composition that is important for seed dispersal (Farwig & Berens, 2012), but the relative abundance of effective and noneffective disperser species. The human‐caused extirpation of some mammals and the drastic changes in abundance of others could be having considerable effects on seed dispersal and ultimate success of many plant species. However, as many trees are long‐lived, the effects on forest structure would only slowly become evident.

Recently, there have been calls for re‐wilding of parts of North America with native fauna (Matthews, 1992), Pleistocene surrogates (e.g., Davis & Moran, 2016; Donlan et al., 2006), and with further biotechnology development, the re‐introduction of de‐extincted species (Sherkow & Greely, 2013). If these plans are undertaken, understanding how these species may affect the ecology of these ecosystems will be important. Studies such as ours that compare paleoecological and modern ecological and evolutionary interactions could provide important information on the wisdom of such future wildlife management decisions.

## Conflict of Interest

None declared.

## References

[ece33008-bib-0001] Barlow, C. (2000). The ghosts of evolution. New York, NY: Basic Books.

[ece33008-bib-0002] Barlow, C. (2001). Anachronistic fruits and the ghosts who haunt them. Arnoldia, 61, 14–21.

[ece33008-bib-0003] Berry, E. W. (1916). The lower Eocene floras of southeastern North America (Vol. 91). Washington Government Printing Office Washington, D.C.

[ece33008-bib-0004] Boone, M. J. , Davis, C. N. , Klasek, L. , del Sol, J. F. , Roehm, K. , & Moran, M. D. (2015). A test of potential Pleistocene mammal seed dispersal in anachronistic fruits using extant ecological and physiological analogs. Southeastern Naturalist, 14, 22–32.

[ece33008-bib-0005] Burton, J. D. (1990). *Maclura pomifera* (Raf.) Schneid. Osage‐orange In Burns R. M. & Honkala B. H. (Eds.), Silvics of North America, Hardwoods. Agriculture Handbook 654 (2, pp. 426–432). Washington, DC: U.S. Department of Agriculture, Forest Service.

[ece33008-bib-0006] Bustamante, R. O. , Simonetti, J. A. , & Mella, J. E. (1992). Are foxes legitimate and efficient seed dispersers? A field test. Acta Oecologica, 13, 203–208.

[ece33008-bib-0007] Chavez‐Ramirez, F. , & Slack, R. D. (1993). Carnivore fruit‐use and seed dispersal of two selected plant species of the Edwards Plateau, Texas. Southwestern Naturalist, 81, 141–145.

[ece33008-bib-0008] Cosyns, E. A. , Delporte, L. L. , & Hoffman, M. (2005). Germination success of temperate grassland species after passage through ungulate and rabbit guts. Journal of Ecology, 93, 353–361.

[ece33008-bib-0009] Côté, S. D. , Rooney, T. P. , Tremblay, J. P. , Dussault, C. , & Waller, D. M. (2004). Ecological impacts of deer overabundance. Annual Review of Ecology Evolution and Systematics, 35, 113–147.

[ece33008-bib-0010] Cypher, B. L. , & Cypher, E. A. (1999). Germination rates of tree seeds ingested by coyotes and raccoons. American Midland Naturalist, 142, 71–76.

[ece33008-bib-0011] Dalke, P. D. , Clark, W. K. , & Korschgen, L. J. (1942). Food habit trends of the wild turkey in Missouri as determined by dropping analysis. Journal of Wildlife Management, 6, 237–243.

[ece33008-bib-0012] Davis, C. N. , & Moran, M. D. (2016). An argument supporting de‐extinction and a call for field research. Frontiers of Biogeography, 8, e28431.

[ece33008-bib-0013] Donlan, C. J. , Berger, J. , Bock, C. E. , Bock, J. H. , Burney, D. A. , Estes, J. A. , … & Soulé, M. E. , (2006). Pleistocene rewilding: An optimistic agenda for twenty‐first century conservation. American Naturalist, 168, 660–681. 10.1086/508027 17080364

[ece33008-bib-0014] Eaton, S. W. (1992). Wild turkey In Poole A., Stettenheim P. & Gill F. (Eds.), The birds of North America (Vol. 22, pp. 1–28). Washington, DC: American Ornithologists' Union.

[ece33008-bib-0015] Everitt, J. H. (1984). Germination of Texas persimmon seed. Journal of Range Management, 37, 189–192.

[ece33008-bib-0016] Farwig, N. , & Berens, D. G. (2012). Imagine a world without seed dispersers: A review of threats, consequences and future directions. Basic and Applied Ecology, 13, 109–115.

[ece33008-bib-0017] Fox, J. F. (1982). Adaptation of gray squirrel behavior to autumn germination by white oak acorns. Evolution, 36, 800–809. 28568235 10.1111/j.1558-5646.1982.tb05446.x

[ece33008-bib-0018] van Geel, B. , Aptroot, A. , Baittinger, C. , Birks, H. H. , Bull, I. D. , Cross, H. B. , et al. (2008). The ecological implications of a Yakutian mammoth's last meal. Quaternary Research, 69, 361–376.

[ece33008-bib-0019] Gill, R. M. A. , & Beardall, V. (2001). The impact of deer on woodlands: The effects of browsing and seed dispersal on vegetation structure and composition. Forestry, 74, 209–218.

[ece33008-bib-0020] González‐Varo, J. P. , López‐Bao, J. V. , & Guitián, J. (2013). Functional diversity among seed dispersal kernels generated by carnivorous mammals. Journal of Animal Ecology, 82, 562–571. 23228197 10.1111/1365-2656.12024

[ece33008-bib-0021] Hackenberger, M. K. (1987). Diet Digestibilities and Ingesta Transit Times of Captive Asian (Elephas Maximus) and African (Loxodonta Africana) Elephants. MSc Dissertation, Department of Animal and Poultry Science, University of Guelph, Guelph, ON.

[ece33008-bib-0022] Howe, H. F. , & Smallwood, J. (1982). Ecology of seed dispersal. Annual Review of Ecology and Systematics, 13, 201–228.

[ece33008-bib-0023] Janzen, D. H. (1982). Differential seed survival and passage rates in cows and horses, surrogate Pleistocene dispersal agents. Oikos, 38, 150–156.

[ece33008-bib-0024] Janzen, D. H. , & Martin, P. S. (1982). Neotropical anachronisms: The fruits the gomphotheres ate. Science, 215, 19–27. 17790450 10.1126/science.215.4528.19

[ece33008-bib-0025] Kistler, L. , Newsom, L. A. , Ryan, T. M. , Clarke, A. C. , Smith, B. D. , & Perry, G. H. (2015). Gourds and squashes (*Cucurbita* spp.) adapted to megafaunal extinction and ecological anachronism through domestication. Proceedings of the National Academy of Sciences, 112, 15107–15112. 10.1073/pnas.1516109112 PMC4679018 26630007

[ece33008-bib-0026] Koike, S. , Morimoto, H. , Goto, Y. , Kozakai, C. , & Yamazaki, K. (2008). Frugivory of carnivores and seed dispersal of fleshy fruits in cool‐temperate deciduous forests. Journal of Forest Research, 13, 215–222.

[ece33008-bib-0027] Lovett, G. M. , Canham, C. D. , Arthur, M. A. , Weathers, K. C. , & Fitzhugh, R. D. (2006). Forest ecosystem responses to exotic pests and pathogens in eastern North America. BioScience, 56, 395–405.

[ece33008-bib-0028] Martin, A. C. , Zim, H. S. , & Nelson, A. K. (1951). American wildlife and plants. New York, NY: McGraw‐Hill.

[ece33008-bib-0029] Matthews, A. (1992). Where the buffalo roam: Restoring America's Great Plains. Chicago, IL: University of Chicago Press.

[ece33008-bib-0030] Meanley, B. (1956). Foods of the wild turkey in the White River bottomlands of southeastern Arkansas. The Wilson Bulletin, 68, 305–311.

[ece33008-bib-0031] Moran, M. D. , Cox, A. B. , Wells, R. L. , Benichou, C. C. , & McClung, M. R. (2015). Habitat loss and modification due to gas development in the Fayetteville shale. Environmental Management, 55, 1276–1284. 25566834 10.1007/s00267-014-0440-6

[ece33008-bib-0032] Murphy, J. L. (2001). Pawpaws, persimmons, and ‘possums: On the natural distribution of pawpaws in the Northeast. North American Archaeologist, 22, 93–115.

[ece33008-bib-0033] Myers, J. A. , Vellend, M. , Gardescu, S. , & Marks, P. L. (2004). Seed dispersal by white‐tailed deer: Implications for long‐distance dispersal, invasion, and migration of plants in eastern North America. Oecologia, 139, 35–44. 14740288 10.1007/s00442-003-1474-2

[ece33008-bib-0034] National Weather Service (2015). Archived Weather Data, Little Rock, AR. Retrieved from Feb. 9, 2017 http://w2.weather.gov/climate/index.php?wfo=lzk

[ece33008-bib-0035] Newsom, L. A. , & Mihlbachler, M. C. (2006). Mastodons (*Mammut americanum*) diet foraging patterns based on analysis of dung deposits In Webb S. D. (Ed.), First Floridians and last mastodons: The Page‐Ladson site in the Aucilla River (Vol. 26, pp. 263–331). Dordrecht, The Netherlands: Springer.

[ece33008-bib-0036] Percy, K. E. , & Ferretti, M. (2004). Air pollution and forest health: Toward new monitoring concepts. Environmental Pollution, 130, 113–126. 15046846 10.1016/j.envpol.2003.10.034

[ece33008-bib-0037] Prasad, S. , Krishnaswamy, J. , Chellam, R. , & Goyal, S. P. (2006). Ruminant‐mediated seed dispersal of an economically valuable tree in Indian dry forests. Biotropica, 38, 679–682.

[ece33008-bib-0038] Robertson, A. W. , Trass, A. , Ladley, J. J. , & Kelley, D. (2006). Assessing the benefits of frugivory for seed germination: The importance of the deinhibition effect. Functional Ecology, 20, 58–66.

[ece33008-bib-0039] Roehm, K. , & Moran, M. D. (2013). Is the Coyote (*Canis latrans*) a potential seed disperser for the American Persimmon (*Diospyros virginiana*)? American Midland Naturalist, 169, 416–421.

[ece33008-bib-0040] Samuels, I. A. , & Levey, D. J. (2005). Effects of gut passage on seed germination: Do experiments answer the questions they ask? Functional Ecology, 19, 365–368.

[ece33008-bib-0041] Sherkow, J. S. , & Greely, H. T. (2013). What if extinction is not forever? Science, 340, 32–33. 23559235 10.1126/science.1236965

[ece33008-bib-0042] Skallerup, H. R. (1953). The distribution of *Diospyros virginiana* L. Annals of the Missouri Botanical Garden, 1953, 211–225.

[ece33008-bib-0043] Taira, S. (1996). Astringency in persimmon In Linskens H. F. & Jackson J. F. (Eds.), Fruit analysis (pp. 97–110). Heidelberg, Germany: Springer, Berlin.

[ece33008-bib-0044] USDA (2016a). Nutritional Nutrient Database for Standard Reference. Retrieved from November 10, 2016 http://www.nal.usda.gov

[ece33008-bib-0045] USDA (2016b). Natural Resource Conservation Service. Retrieved from November 10, 2016 https://plants.usda.gov/plantguide/pdf/pg_divi5.pdf

[ece33008-bib-0046] Van Weyenberg, S. , Sales, J. , & Janssens, G. P. J. (2005). Passage rate of digesta through the equine gastrointestinal tract: A review. Livestock Science, 99, 3–12.

[ece33008-bib-0047] Willson, M. F. (1993). Mammals as seed‐dispersal mutualists in North America. Oikos, 67, 159–176.

[ece33008-bib-0048] Worth, C. B. (1975). Virginia opossums (*Didelphis virginiana*) as disseminators of the common persimmon (*Diospyros virginiana*). Journal of Mammalogy, 56, 517.

