# Supplementary figures and images for: Seed dispersal of Diospyros virginiana in the past and the present: Evidence for a generalist evolutionary strategy
Source: Ecol Evol. 2017 May 4;7(11):4035–43. doi: 10.1002/ece3.3008 (PMC5468125; doi:10.1002/ece3.3008)

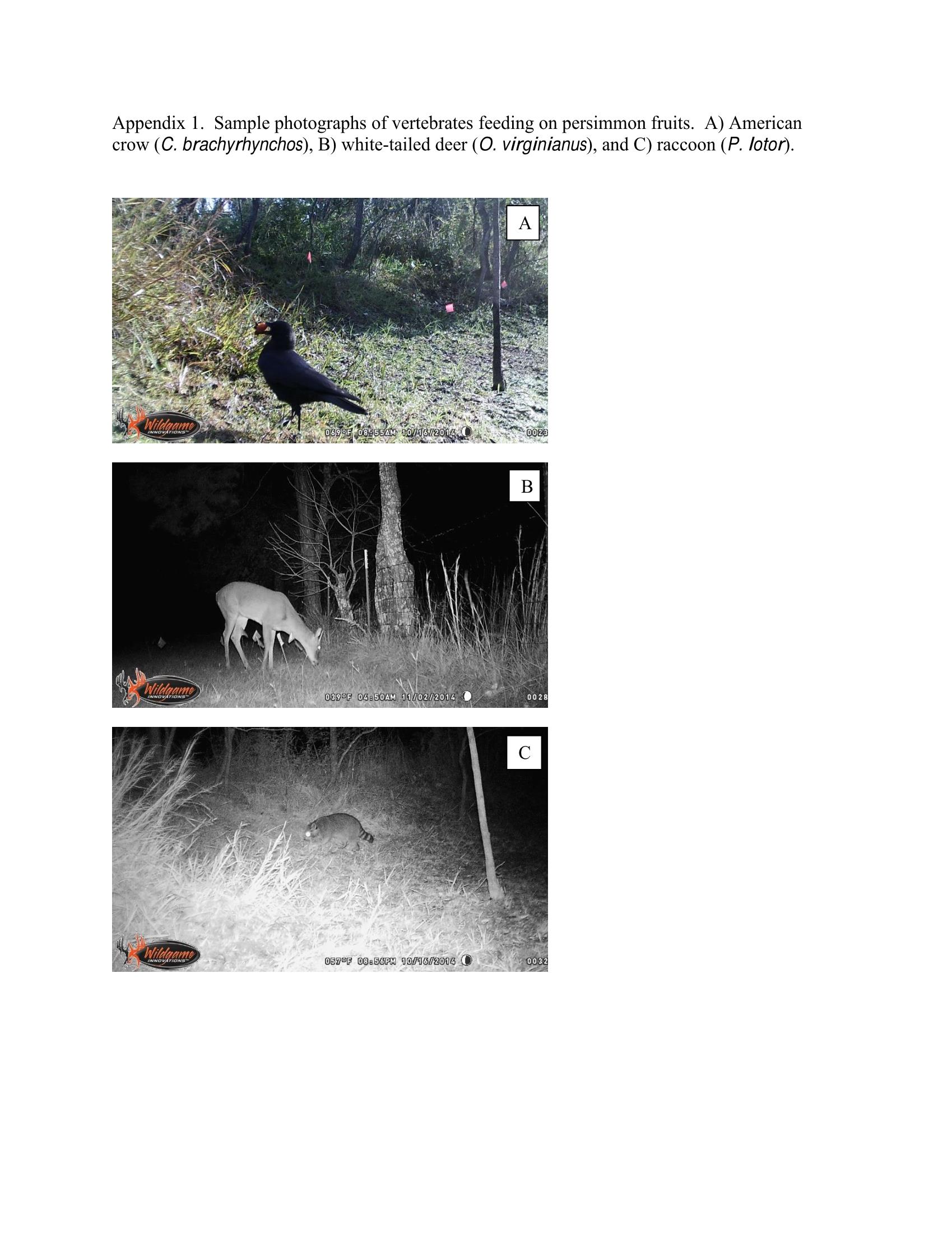

Supplement: Supplementary file 1 [file ECE3-7-4035-s001.jpeg]
